# From their own perspectives: a qualitative study exploring the perceptions of traditional health practitioners in northern Uganda regarding cancers, their causes and treatments

**DOI:** 10.1186/s12875-021-01505-w

**Published:** 2021-07-19

**Authors:** Amos Deogratius Mwaka, Jennifer Achan, Winnie Adoch, Henry Wabinga

**Affiliations:** 1 Department of Medicine, School of Medicine, College of Health Sciences, Makerere University, P.O Box 7072, Kampala, Uganda; 2 School of Public Health, College of Health Sciences, Makerere University, Kampala, Uganda; 3 Kampala Cancer Registry, Department of Pathology, School of Biomedical Sciences, Makerere University, Kampala, Uganda

**Keywords:** Traditional medicine, Complementary medicine, Therapy, Traditional health practitioners, Uganda, Cancer

## Abstract

**Background:**

Many cancer patients in the low- and middle-income countries seek care with traditional health practitioners (THPs) and use traditional and complementary medicines (T&CMs) for treatment of cancers. Little is known about the perceptions and influence of THPs on cancer patients’ help-seeking and treatment decisions. We aimed to explore perceptions of THPs regarding cancers, cancer causes, and preferred treatments for cancers, in order to identify aspects that can inform interventions to improve cancer outcomes in Uganda.

**Methods:**

We conducted this ethnographic study in northern Uganda. In-depth interviews were conducted at the respondents’ homes in quiet, open places, and in the absence of none- respondents. Interviews were audio-recorded and transcribed verbatim within a week of the interviews. Thematic qualitative analysis approaches were used to identify themes and subthemes.

**Results:**

We included 21 respondents in the study; most were male (16/21), married, with median age of 59 years (range 39 – 80). Most respondents perceived cancer as a new and challenging disease, while one respondent thought of cancer as a result of an imbalance within the body. Most confessed unawareness of the causes of cancers, but believed that cancer could result from the interplay of a number of factors including poor diets, ingestions of chemical agents, and assaults by the spirits of the dead. Some reported that cancers (especially of women’s genital tracts) were sexually transmitted, or caused by accumulation of dirt. Only few healers treated cancers. Most respondents reported that they referred cancer patients to biomedical facilities, sometimes after they have first used their medicines. Most respondents hoped that collaborative research with scientists could help them identify potent T&CMs that cure cancers.

**Conclusion:**

Traditional health practitioners require training on cancer causes, symptoms and signs, and the necessity for prompt initiation of effective treatments in order to improve cancer outcomes. The predisposition of the majority of respondents to refer cancer patients to biomedical services sets a fertile ground for meaningful cooperation between biomedical and traditional health practices. The national health system in the low- and middle-income countries could formally recognize traditional health practices as a component of the national healthcare system, and encourage the two to practice side by side.

**Supplementary Information:**

The online version contains supplementary material available at 10.1186/s12875-021-01505-w.

## Background

Cancer patients worldwide use Traditional & Complementary Medicines (T&CM) before and after cancer diagnoses, as well as concurrently with conventional cancer therapies [1]. A systematic review of studies on T&CM use among cancer patients during 2009 to 2018 showed an overall prevalence of use of 51% [2]. Similarly high prevalence of use of T&CM was revealed in a systematic review and meta-analysis of 155 studies involving 809,065 patients from all around the world. The study showed that 22% of cancer patients use herbal medicine during their cancer disease. Use of T&CM was highest in Africa (40%; 95%CI: 23—58) and Asia (28%; 95%CI: 21—35). The pooled prevalence of use was lower across the high-income countries (17%; 95%CI: 14—21) [3]. Use of T&CM is common among cancer patients from both the low- and middle-income countries as well as the high income countries (HIC). A systematic review study showed that the use of T&CM among indigenous population across the USA, Australia, New Zealand and Canada ranged between 19% to 57.7% [4]. In the USA, a large national cancer database study showed that 20% (258/1032) of cancer patients use complementary therapies; most of the complementary medicine users were likely to refuse conventional cancer therapies especially radiotherapy, and experience poorer 5-year survival [5]. Still in the USA, an analysis of data obtained from the National Health Interview Survey of 2012 showed that 33.3% of 3118 cancer patients and cancer survivors used T&CM in the past 12 months [6]. In Canada, the use of complementary therapies, especially biologic agents increased from 15.6% (*N* = 141) before cancer diagnoses to 51.8% after histology diagnoses [7]. Similarly high prevalence of use of T&CM have been reported among cancer patients from Europe. In a population-based survey in northern Norway, 33.4% of 404 participants who reported having a cancer used some form of T&CM before and concurrently with conventional cancer medicines, and 13.6% had consulted a traditional healer for their symptoms [8]. In Sweden, 34% (256/755) of cancer patients used complementary therapies; 26% used them after cancer diagnoses [9]. In Italy, a multicenter study showed that 48.9% of 468 cancer patients used or were using complementary therapies [10]. In Hungary, 52.6% of 135 breast cancer patients used complementary therapies before diagnosis, while 84.4% used the therapies during conventional cancer therapies [11]. At an academic center in Switzerland, 56% of 132 cancer patients had ever used T&CM since cancer diagnoses; 45% were using the remedies concurrently with conventional medicines during the survey [12]. Use of T&CM for the treatment of cancers and cancer symptoms was also found to be common in the Middle East and Asia. In Saudi Arabia, 69.9% of 156 cancer patients attending care at a cancer treatment facility self-reported use of T&CM [13]. The prevalence of T&CM use among cancer patients in Turkey ranged from 54.5% to 61% [14–17]. In Malaysia, 41.6% (184/393) to 61.2% (74/121) of cancer patients used some kind of T&CM for the treatment of their cancers [18, 19]. In Taiwan, analysis of a national database on cancer patients showed that 12.8% (*n* = 74,620) used T&CM, and use varied with cancer sites [20]. A review of studies from the low-income and lower middle-income countries showed that 54.5% of cancer patients used T&CM; 26.7% used T&CM concurrently with conventional cancer therapies [21]. A systematic review on the use of T&CM in sub-Saharan Africa among cancer patients showed that 65% to 79% used the remedies during their cancer disease [22]. Similarly, a scoping review of studies from sub Saharan Africa showed that 60.0% of cancer patients used T&CM concurrently with conventional cancer therapies [23]. In Tunisia, 85% (102/120) of cancer patients at a specialized cancer treatment facility self-reported using T&CM for the management of their cancers [24]. In Tanzania, 88.1% of 160 cervical cancer patients attending care at the Ocean Road Cancer Center reported concomitant use of T&CM [25]. In Uganda, 55.4% (240/434) of cancer patients with solid tumors attending care at the specialized national cancer treatment center self-reported using T&CM for the treatment of their cancers [26].

In Uganda and Ethiopia, women with possible symptoms of breast and cervical cancer often prefer to first seek care with the traditional health practitioners (THPs) before presentations at biomedical facilities [27, 28]. The use of T&CMs is therefore common in sub Saharan Africa, and is likely to continue alongside conventional cancer treatments [29]. We have therefore used the theory of medical pluralism to inform our investigation regarding perceptions of cancers, cancer causes, and preferred treatments among the THPs. This theory recognizes that conventional biomedicine and traditional and alternative health practices can coexist in harmony rather than in opposition [30, 31]. While integration fosters double standards for evaluating, judging and setting decision making principles for conventional and unconventional medicines, ignores the inherent and rather irreconcilable differences in the philosophical basis, beliefs and practices of the two health systems, medical pluralism on the other hand recognizes the values inherent in both systems and offers opportunity for rationale choice, thereby upholding the ethical principle of autonomy for the patients [30]. Patients ought to make their choices regarding sources of help-seeking and treatments. Physicians’ paternalistic tendencies that have for long been part and parcel of biomedicine maybe moderated by the official recognition of medical pluralism that promotes autonomy [32, 33]. Autonomy, not paternalism ought to underpin ethical healthcare practices in the twenty-first century [34–37]. The proliferation of medical information on the internet has not only undermined paternalism and prompted autonomy, but indeed blends with the principle of medical pluralism [32].

Recognition of medical pluralism is critical since the use of T&CM is not likely to stop any soon because there are several reasons cancer patients in sub Saharan Africa use T&CMs, including to: cure/treat cancers, manage cancer symptoms, boost the immune system, and improve physical and psychological wellbeing [23, 26]. Other patients prefer to use T&CMs due to fear of surgery, dissatisfaction with conventional medical care, multiple side effects of conventional cancer medicines, recommendation by friends and family, trust in T&CM providers, or because T&CMs are readily available and cheaper than conventional medicines [38–44]. Patients may also prefer T&CMs because the practitioners often provide payment schedules that are favorable to the patients [45, 46]. Some cancer patients seek care and use T&CMs as part of the process of search for therapies that are in line with traditional values and norms, and their philosophy of life about health and illness [43, 47, 48]. In addition, unlike biomedical practitioners who are perceived by patients as laid back and unconcerned about patients’ lives beyond the disease they are managing, THPs also deal with the social realities of the patients and provide comfort to the patients and their families [49, 50]. There is evidence that THPs in Uganda influence help-seeking decisions for chronic diseases such as mental health, diabetes, and tuberculosis [51–53]. In addition, there is also evidence that seeking care with THPs and using T&CMs may be harmful for cancer patients. For example, consultations with THPs potentially delay health-seeking and lead to advanced stage cancer at diagnosis [43, 54]. It has also been reported that THPs sometimes engage in practices that could lead to delay in presentation and diagnoses, and raising unrealistic expectations of cure where none maybe possible [55–57]. Nevertheless, THPs have remained popular among their people: they often have great credibility and esteem in their communities. People listen to them and take seriously what they communicate [57]. They seem to understand the behaviours, values and language of their communities. They are accepted and have remained central in health services possibly because they provide care within the patients’ cultural context and belief system [58, 59]. In South Africa, THPs have been shown to have some basic knowledge of cancer; for example, they considered cancer diagnoses in patients with sores that do not heal easily, lumps, abnormal bleeding, and discharge. The THPs were also aware of some risk factors for cancers including smoking, use of alcohol and early sexual intercourse [60]. Therefore, THPs could be harnessed for health education about cancer prevention and practices, and to promote early detection and diagnoses of cancers. However, outside South Africa, little is known about THPs’ understanding of the development, causes, and treatments of cancers. There is also limited awareness of whether THPs refer or dissuade patients with possible cancer symptoms from seeking care at biomedical facilities. In this study we explored the perceptions and beliefs of THPs in northern Uganda about cancers, cancer causes, and preferred treatments for cancers. This knowledge could inform the national healthcare system’s choice regarding integration of traditional health practices with biomedicine or adoption of medical syncretism or pluralism, thereby recognizing and supporting the two systems to work side by side.

## Methods

### Study design and setting

We conducted an ethnographic study involving in-depth interviews and observations of the healers’ practices and medicines. However, not all the healers accepted us to witness their practices and or view their concoctions. The qualitative approach, often used to understand a research problem from the respondents’ own perspectives, allows for an in-depth exploration of a topic about which little is currently known [61]. The approach attaches importance to meanings, views, and experiences of respondents [62, 63]. This study was conducted in northern Uganda involving respondents from all the eight Acoli districts. The people in this region are predominantly Nilotic cultural group [64, 65]. The region experienced a 20 year-long (1987 to 2006) armed conflict that displaced more than 90% of the population into internally displaced person’s camps, and devastated the healthcare system [66]. People in the region commonly use T&CMs for various ailments including cancers [67]. This is in spite of the fact that in Uganda, the mainstream national health system does not include THPs and T&CMs. Patients who use T&CMs seek them outside the mainstream healthcare system and pay for them out of their pockets. The national health system of Uganda comprises the public (governmental facilities), private-for-profit (PFP), and private not-for-profit (PNFP) facilities. The public health system ranges from health center (HC) I to the National Referral Hospitals. Health Center I provides health promotion and preventive health services mainly by village health teams (VHT), and has no physical buildings. Health Center II has a physical structure and provides outpatient curative, preventive and health promotion services. In addition to the services provided at HC II, Health Center III provides inpatient, limited laboratory services, and maternal child health services including deliveries and admissions. Health Center IV provides inpatient, emergency surgical and laboratory services in addition to services provided at HC III. The next level of care is the district general hospitals that provide additional care including radiology, and higher level laboratory and surgical services. The tertiary and super-specialized care services are provided at the Regional Referral and National Referral Hospitals [68]. However, in additional to the national health system, there exists the traditional and complementary medicine services of various kind and grades. There is therefore some elements of medical pluralism in Uganda; the practice of healthcare involving services and services providers with different and sometimes divergent philosophies, ethics, and methods of practices [30, 31].

### Study population and period

We recruited THPs and cultural leaders in charge of healing rituals, approaching men and women aged 18 years and above who resided in the 8 study districts, and were registered with the Acoli Cultural Institution (Ker Kwaro Acoli) as traditional health practitioners, or identified by clan chiefs as leaders of healing rituals. Unregistered healers were identified through snowballing approach by the registered healers [69–71]. In this sub study, we include data from the THPs. Data collection for the study was conducted from January to May 2018.

### Sampling and recruitment of respondents

We used purposive sampling to recruit respondents from each of the 8 districts. We obtained a list of THPs registered with Ker Kwaro Acoli (KKA) from the office of the Prime Minister of KKA in Gulu. We also inquired from the traditional chiefs and local council leaders about people regarded as THPs in the study districts. We selected those who were mentioned by at least two local council leaders and chiefs of the areas, approached them by phone, and set appointments for interviews. Upon acceptance to participate, the respondents were provided detailed information on the study objectives, inclusion criteria, consent procedures, and the potential use of the information to be gained from the study. All respondents provided written informed consents before interviews. We obtained additional verbal consents from respondents to audio-record the interview proceedings.

### Data collection

Data were collected using an interview guide developed from literature on T&CM use and views of THPs in Africa and Asia, and guided by the research questions. The guide was piloted with three THPs. The research assistant transcribed the pilot data which was thereafter analyzed manually. The guide was refined on the basis of this data (Additional file 1). The main questions that generated data for this sub study were: 1. Kindly share with us your opinion regarding use of traditional health practices in treatment of cancers especially breast and cervical cancers. 2. If you consider the use of traditional health practices as useful in treatment of cancers, please tell us some of the benefits of using these remedies in treating cancers. 3. Please tell us some of the traditional and complementary medicines (the names and how they are used – roots, leaves etc.) that are used in the treatment of cancers. 4. If you consider traditional remedies as not useful or even harmful in treatment of cancers, especially breast and cervical cancers, please share with us what you consider as harms or disadvantages of using these remedies in treatment of cancers. 5. What are cancers called in Acoli language (names in Luo)? 6. What causes cancer? 7. Specifically tell us the things that cause breast and cervical cancers? (Thematic area 7 of the study guide).

Interviews were conducted by two research assistants in the respondent’s home, in the language of choice of the respondent, and in the absence of any none-respondents to avoid interference. All the interviews lasted between 60 and 120 min, and were audio-recorded. The research assistants also made field notes on nonverbal communications, which were used to supplement the audio-recorded information during transcriptions to ensure completeness of data. JA supervised the process of data collection and participated in data transcriptions and translation together with the male research assistant.

### Data management and analysis

The research assistants transcribed the interview recordings verbatim within a week, when field interactions were still fresh in their minds. ADM listened to two randomly selected interview recordings while reading through the respective transcribed data to ensure completeness and accuracy of the transcriptions. ADM also reviewed the field notes to capture the fullness of the field experiences and immerse himself in the data before and during analyses. ADM developed analysis codes based on line-by-line reading of four transcripts. The final set of codes (Table 1) were agreed on through consensus between the investigators and mentors. The codes included descriptions/definitions of cancer, perceived causes of cancer (risk factors), symptoms and signs of cancers, preferred treatments, perceived benefits and side effects of the treatments, how a person becomes a THP, and the perceived future of traditional health practices. ADM conducted data analysis through an iterative thematic analysis process [72], supported by ATLAS.ti version 6.1.1. He applied codes (Table 1) to all the corresponding meaning segments in each transcript. The meaning segments were retrieved, aggregated and used to formulate themes and subthemes. Representative quotes were used to exemplify each theme in the results section, accompanied by salient socio-demographic characteristics including respondent’s number, sex and age group.

**Table 1:** Analyses codes and their definitions

Codes	Code definitions
**Definition of cancer**	Any references to what cancer is, or is not
**Help-seeking for self and family**	Any references to where the healer and family members go for help-seeking whenever illness is perceived
**Medical pluralism**	References to whether sick people should seek help from several sources including biomedicine and traditional health practices concurrently or sequentially, and whether such practices are considered good or harmful to the patients
**Causes of cancer**	Any references to what leads to development of cancer or how cancers are perceived to come about in a person
**Symptoms of cancers**	Any references to what are perceived as indicators or signs that a person with cancer develops
**Diagnosis of illness**	References to how a healer or a person may detect a particular kind of illness including cancers, and give names or labels to the illness
**Treatments for cancers**	Any references to concepts about the types of treatments or remedies that are considered to work on cancers, including why it is thought so
**Forms of healing**	References to the different approaches and or things that THPs do to help patients regain health, and the types of healing practices in traditional medicine practice, including giving medicines, surgery, incantations, and administration of rituals
**Rituals used in treatment**	All references about things that healers or elders do other than giving physical medicines, to help a person who is ill to get better or regain health
**Illnesses treated**	References to the types of diseases or illnesses that a healer says he/she treats and how he/she decides which illnesses to treat or specialize in treating
**Becoming a traditional health practitioner**	Any references to how one becomes or became a THP, including the processes of initiation and or source of inspiration to become a healer
**Power of healing**	References to the ways in which one gets inspirations, desire to heal, and actually be able to help a sick person regain health
**Types of THPs**	References to the different categories of healers based on types of services provided or how they treat illnesses and or how they became healers
**Duration of practice as THP**	References to the period since when a THP gained healing power and started providing help to people to regain health
**Detection of potent medicines**	References to how a THP gets to know which plants/materials work as medicines for particular ailments
**Awareness of good THPs**	References about how members of the community get to know who a good THP is and therefore decide to seek help with him/her
**Occupation**	Any references to what the THP used to do before becoming a healer or does concurrently with healing practices, including any formal qualifications
**Traditional surgical practices**	Any references to practices that require cutting on a person perceived as ill, with the intention to relieve distress, pain or cause healing, including the title given to the person who does the cutting procedure
**Safety in surgery**	Any references to the conduct of traditional surgical practices in a way that prevents dirt and infections from getting into the patient, including how to clean and sterilize the equipment used in the surgery
**Cancer early detection**	Any references to whether there are stages that cancers develop through, what needs to be done in order to know that there is cancer when it is still in early stage, how early detection can be done, and the benefits for early detection
**Sources of ill health or diseases**	Any references to what leads people to fall sick or how illnesses get into people and therefore make them become sick
**Challenges treating cancers**	References to difficulties experienced in treating patients with cancers and reasons for such difficulties
**Cancer prevention**	Any references to what should be done to reduce the chance of a person getting a cancer/cancers, and whether it is difficult or easy to prevent cancers
**Collaborations between HCP and THP**	All concepts related to whether or not THPs and biomedical practitioners should work hand in hand with each other including referring patients to each other’s practices, and reasons for such referrals
**Meaningful collaborations**	References to the conditions that need to be put in place, or things to be done so that THPs and biomedical practitioners can work together in harmony with mutual benefits in the prevention and management of cancers
**Hindrances to progress in THP practices**	Perceptions and references to what makes the field of traditional health practice not develop, grow, flourish or be practiced in the open, including the difficulties that THPs experience
**Self-protection during THP practice**	References to precautions undertaken by THPs to avoid getting infections from their patients

*THP* Traditional Health Practitioners, *HCP* Healthcare Practitioners

## Results

### Respondents’ characteristics

The study included 21 respondents, aged 39 – 80 years. The median age was 59 years. Most of the respondents were male, and married. Majority had either attended no formal education or did so only up to primary level (Table 2).

**Table 2:** Respondents’ Characteristics

Characteristics	Respondent’s code	Total
**Sex**		
Male	R2, R3, R4, R6, R7, R9, R10, R11, R12, R13, R14, R15, R16, R17, R18, R20	16
Female	R1, R5, R8, R19, R21	05
**Age group (Years)**		
30—39	R1	01
40—49	R8, R9, R10	03
50—59	R2, R11, R12, R13, R14, R15	06
60—69	R16, R17, R18, R19, R20, R21	06
70—79	R3, R4, R5	03
≥ 80	R6	01
Missing	R7	01
Median age: 59		
**Marital status**		
Married		21
**Educational attainment**		
No formal education	R3, R7, R13	03
Primary education	R1, R4, R5, R6, R12, R14, R19, R21,	08
Secondary education	R8, R11, R17	03
Advanced level education	R15	01
Tertiary/university	R9, R18	02
Vocational training	R10, R16, R20	03
Missing	R2	01
**District of residence**		
Agago	R3, R20	02
Amuru	R2, R5, R7	03
Gulu	R9, R10, R14	03
Kitgum	R11, R16, R19	03
Lamwo	R4, R6, R12	03
Omoro	R1, R21	02
Nwoya	R13, R17	02
Pader	R8, R15, R18	03

Themes that emerged from the data included (i) respondents’ understanding of cancer as a new and challenging disease, a form of an imbalance in the body; (ii) perceived causes of cancers e.g. ingestion and accumulation of chemical agents in the body, poor diet, heredity, and poor genital hygiene; and (iii) perceptions and beliefs regarding use of T&CM in treatment of cancers.

### Understanding of ‘cancer’

Most respondents described cancer as a new and challenging disease, and as an imbalance within the body. They said cancer does not have any particular name in Acoli.

#### New and challenging disease

Most respondents concurred that cancer was unknown to the Acoli people. Names for illnesses were given by the ancestors, but they were not aware of a name of any disease in the past that corresponds to what cancers are known to be today.

“In the past we didn’t know them but there is a possibility that it has always been there. Culturally I don’t know of any explanations for cancer”, **(R8, Male, 40-49 years)**.

“You should know that cancer is a new disease in this part of the country and even traditional medicine practitioners can’t understand it. […]. And it comes in various forms, sometimes it catches the breasts, sometimes it comes with blood flow”, **(R9, Male, 50-59 years)**.

#### Imbalance within the body

One respondent, with a diploma in health sciences, described cancer as a form of imbalance within the body that only becomes manifest with time, especially when certain circumstances have put stress onto the person. He underscored that everyone has cells that could potentially develop into cancers, although not everyone develops cancers.

“I would say it is not a sickness but an imbalance within the body which causes conditions which come up like cancers; and some are reversible. Specifically let me talk about cancer, we have cancer cells in our bodies, but when time is favorable that is when they come up”, **(R20, Male, 60-69 years)**.

In summary, traditional health practitioners have a limited understanding of cancer as a disease. The practitioners derive their knowledge of diseases and their treatments from the cultural knowledge repertoire. Therefore, the traditional health practitioners become challenged once the presentations of diseases or disorders do not conform to any known diseases previously described by their predecessors.

### Perceived causes of cancers

Most respondents admitted limitations in knowledge regarding the actual causes of cancer. Majority found it difficult to articulate the causes of cancers because their ancestors never encountered diseases of that nature and therefore their mentors who initiated them into traditional health practices did not pass this knowledge to them.

“The way cancer starts, what brings and how it starts showing in you is a mystery; but you only get to realize pain in your body, and that pain only comes when it has already existed in your body for quite long; it is only the medics in the hospital who can diagnose that it is cancer, no one should boast that they are the ones who diagnose it. I also remain in the same position. I also don’t know what brings cancer, and for me I started treating it after it had already come”, **(R7, Male, Missing age)**.

“But God has created people differently in this world; but at times people think it is because someone with ill intentions has bewitched them or they have been curse but that is not the case. We simply don’t know what causes cancer and how to cure it”, **(R16, Male, 60 – 69 years)**.

The few respondents who suggested some perceived causes of cancers held varying opinions, mainly speculative. Reported causes depended on traditional beliefs, and knowledge acquired from biomedical sciences. The perceived causes included:

#### Chemicals agents

A substantial minority of respondents said that cancers develop slowly, and could result from prolonged exposures to and accumulation of chemical agents and toxins found in workplaces including factories.

“Actually the causes of most cancers are unexplainable even to the Doctors; however some are explainable. For example, if you have been working in a chemical factory for long you will have strange sicknesses which can’t even be diagnosed in the hospitals. At times you can ask the patients and they tell you that yes they have worked in a factory for long, so you just know that this thing has been caused by working in the factory. You see, so it doesn’t come in one day, it comes slowly, **(R6, Male, 80+ years)**.

#### Metal deposition in the body

In traditional Acoli society, women would grind millet and sorghum, and sesame seeds and ground nuts on stone slates. The respondents thought those were healthy habits because the stones were natural and so even if they wear out into the food, there would be no harmful consequences. However, nowadays people use steel and other iron products to grind these substances. Saucepans and utensils are now scrubbed using steel wires unlike in the olden Acoli society when women would use specially prepared sponges from grass and tree-barks. Respondents thought that cancers could result from the accumulation of the residues from the metallic substances that go into the food.

“The thing that brings cancer . . . Some people have been mentioning that use of steel wire; some people guessed that grinding mills for grinding flour. But I also thought deep and get to question that people have lived many years ago and I personally found grinding mills in existence, and right now I am old but still alive and strong; so if steel wire I may believe. When you scrub your saucepan neatly and you dry it, pick the saucepan and wipe the inside with your fingers; you will still find some dust from steel wire and this is how it enters into our bodies, mix with fluid and ends up causing cancer in our bodies. I can accept that cancer comes in that way”, **(R7, Male, Missing age)**.

The metallic residues are thought to settle in and block blood vessels and ducts in the body. The blockage causes back pressure to develop and subsequent rupture of the blood vessels and ducts, leading to wounds – the hallmark of cancers.

“Yes, breast cancer is like you who swallows food and takes water, then it tries to get out of the nipple but get stuck in the breast, because there is a sieve in the breast which will arrest it. It is the steel wire that I have been talking about. […] For the cervix, it is also the same; it will go and settle, once it settles, it has to bring wound and once it has come, it will increase further in size . . . this is my suggestion, **(R7, Male, Missing age)**.

#### Poor diet

The respondents also thought the nature of the food consumed these days lead to development of several serious illnesses including cancers. The respondents reported that in the olden Acoli society food were eaten fresh from the gardens; oils were extracted from naturally grown foods including ground nuts and sesame seeds. These foods were considered nutritious and safe, unlike foods of these days that are bought from the markets; the sources of the foods, handling and conditions of storage are not known. Many people handle the foods, and some could be evil-handed.

“I think cancer is caused by poor eating especially the foreign foods that we have today. Food that the Acoli are not used to is also causing cancer. I leave that to the doctors to investigate”, **(R11, Male, 50 – 59 years)**.

“One of the main causes of cancer today is the food we eat because we eat toxic foods . . . That means we have to come up with good food, with organic food . . . those organic foods can suppress those cancer cells. In Acoli, they don’t want you to eat fresh meat; you first smoke it or hang it somewhere so it drips first”, **(R20, Male, 60 – 69 years)**.

#### Cancers are hereditary

A few respondents reported that some cancers are inherited from parents. They based this deductions on their observations of circumstances where more than one person in a family or clan have developed particular cancers.

**“**Cancers also run in certain families. I know of a family that has had almost three people who have had cancer. The latest is their sister who has breast cancer. So I think it runs in the family”, (**R8, Female, 40 – 49 years)**.

#### Bad spirits

One respondent asserted that cancers are caused by bad spirits. These are often spirits of clansmen who have not found rest among the living dead for several different reasons. These spirits roam around, attack people and cause various ailments including cancers. In Acoli, several rare and strange illnesses have been attributed to these vengeance spirits.

“Those are spirits (*tipu*). They are spirits that when treated well can heal. It is just that they are very few that come to the healers when they are sick”, **(R18, Male, 60 – 69 years)**.

#### Cancers are sexually transmitted

Most respondents considered that some cancers, especially those affecting the reproductive organs and genitals are sexually transmitted from person to person, and or due to poor genital hygiene. In particular, cervical cancer and other cancers of the female genital tract are thought to be due to intra-vaginal accumulation of dirt or poor vaginal hygiene.

“The root cause and main cause is some of the girls go for what they are not supposed to do, and they acquire it. It can be transmitted through sexual intercourse or sharing pit latrine or getting into contact with somebody who is already infected”, **(R8, Female, 40 – 49 years)**.

“So you know like women are open; when you go to a toilet, you can get things like candida or syphilis. Those can accumulate in you and become things like cervical cancer, you see”, **(R8, Female, 40 – 49 years)**.

In conclusion, majority of respondents were quite uncertain and baffled about the causes of cancers. However a substantial minority reported that cancers are due to lifestyle changes and exposures to recent environmental toxins and foreign diets that are considered unhealthy compared to the traditional dishes. Some respondents considered that cancers are heredity or perhaps due to shared exposures among family members who tend to develop particular cancers. Most of the articulated perceived causes are based on observations rather than knowledge acquired from the cultural repertoire or knowledge passed on from predecessors.

### Treatment of cancers with traditional and complementary therapies

#### Adjunctive role of T&CMs in healing

Treatment with T&CMs were considered essential for the treatment of most serious illnesses. Most THPs would recommend T&CMs before or during treatment with conventional medicines because T&CMs are natural products and have limited side effects; they have cleansing effects on the individual, thereby priming him/her for better response to conventional cancer therapies.

“The benefit is really great . . . herbs don’t have side effects unlike the modern medicines. Our herbs can even be eaten as food unlike the modern drugs”, **(R1, Female, 30 – 39 years)**.

Most THPs reported that healing requires a complete equilibrium in the life of the patient. Even if a conventional medicine is effective, the patient will not heal if he/she is not at peace with the ancestors in the world of the living dead. The equilibrium is brought about by conscientious use of T&CMs and rituals. If the perceived moral wrong is very serious, then rituals are recommended to cleanse the patient and make them ready for conventional medicines.

“The way I understand most illnesses, when the healthcare professionals try in vain to treat it, they send you back home so you seek traditional remedies to it. If you are done with such traditional ways of treatment then when you go back, the modern medicines will work. […] When you first sort out the traditional issues, then the modern medicines will now work and you will live”, **(R15, Male, 50 – 59 years)**.

#### No T&CMs cure cancers

Most respondents did not treat cancers mainly because they do not have medicines that cure cancers. They do have medicines for diseases known to their ancestors.

“No, I did not. She was taking medicine that they gave her from the hospital so I couldn’t interrupt her treatment and beside I don’t have its medicine. And I have not dreamt about it, maybe one day when I dream then I will treat it”, **(R5, Female, 70 – 79 years)**.

“You should know that cancer is a new disease . . . That is why there is no one in the community who says they can give medicine for cancers, no one should try treating it. It is even defeating the white people, and they say that if it takes long it can’t heal completely . . . So, I think more research should be carried out in that field to try and come up with its cure. In the past elders would do anything to try and remedy certain illnesses but this is beyond us now. I simply can’t explain it. And my plea to you is as you continue moving don’t let anyone deceive you that they can treat cancer, it is a lie”, **(R9, Male, 40 – 49 years)**.

Most THPs reported referring patients with cancers to their colleagues who treat cancers or refer the patients to biomedical facilities.

“You see in this work of ours, one has to be honest to themselves and also to the patients. If I can’t explain the cause of a disease or I feel I am not the right person to handle it, then I refer them to someone else who can do it more than me. So when I see a rare illness that I have not seen before, I refer them to the hospital for tests, and I believe cancer falls in that category”, **(R8, Female, 40 – 49 years)**.

#### Medicines for the treatment of illnesses with similar presentations can be used

The discovery of potent medicines in the Acoli Traditional Medicine system takes the trajectory of enlightened trials based on the philosophy of similarity; illnesses with similar symptoms respond to similar medicines. Healers would then evaluate the symptoms of a disease, compare them with known illnesses with known medicines/treatments. The healer then selects from among those treatments and tries them on the new disease without known treatment/cure. Appropriate modifications are conducted by adding various components as necessary.

“You know cancer wasn’t there in the past; it is a new phenomenon . . . For example, cervical cancer presents like syphilis; so to us herbalists, we put it in that category. We use medicines that is similar to the one we use to treat syphilis. You see, especially cervical cancer, it affects the opening of the womb and spreads to the lower belly. So if we give that medicine and she starts taking, all that will come out”, **(R1, Female, 30 – 39 years)**.

#### T&CMs cure cancers

A substantial minority of respondents (3 respondents) said they have medicines that treat and cure cancers. They provided testimonies of successfully treating patients with confirmed cancer diagnoses. One respondent claimed to possess medicine that prevents cancer development and or progression.

“So, with cancer cases we need to affect it from the very roots. That is what this medicine can do. I have treated very many cases of cancers; breast cancer, cervical cancer, colon cancer. This medicine you don’t only take it when you are sick, you can also take it to cleanse up. So you can keep, you can always take it when you need it” **(R20, Male, 60 – 69 years)**.

“Cancer treatment using traditional medicine is the best, especially if the patient is brought in its early stages. It does not have side effects like falling off of hair or nails”, **(R21, Female, 60 – 69 years)**.

However, the THPs who treat cancers have potential to delay health-seeking by providing advice that would require some particular treatments before seeking care at biomedical facilities.

“I told them that this is cancer and this cancer when it is operated this boy is going to die. He should first be on my medication for even a year if they want this thing to be removed so that he regains energy. This people never listened to my advice. Hearing that it is cancer, they went and tested and found out that it is indeed cancer . . . They had money, so they moved to the hospital; from there they paid a lot of money and they operated the boy. They took him to India and he died, because the machines which were being used on him alone was a problem. They would have listened and let me detoxify”, **(R8, Female, 40 – 49 years)**.

In this study, most of the THPs would not treat cancers except three. This is mainly because they did not inherit potent medicines from their predecessors, nor have they dreamt about traditional medicines that cure cancers. The THPs reported that they sometimes try to treat cancers with T&CMs used for treatment of diseases that present like the particular cancers and or affect the same parts of the body like the cancers. Majority of the respondents have however not observed any durable positive response of cancers to T&CMs and would therefore refer patients they consider to have cancers to their colleagues who treat cancers and or to the biomedical facilities.

## Discussion

Traditional health practitioners (THPs) described cancers as a new and challenging disease, an imbalance in the functioning of the body, which is poorly understood both by the traditional health system and biomedicine. They admitted limitations in their knowledge regarding the causes and appropriate treatments for cancers. Most respondents said that cancer could be caused by various things including exposure to and accumulation of chemical agents and metallic residues in the body, poor diets, heredity, and vengeance spirits of the dead who have not found peaceful resting places in the world of the living dead. Women’s genital cancers including cervical cancer were thought to be sexually transmitted and or result from accumulation of dirt in the woman’s genital tract. The majority of respondents did not know T&CMs that can be used for treatment of cancers, and did not engage in treating cancer patients. A substantial minority treated cancers with combinations of herbal preparations that were originally crafted for treatment of diseases that the cancers resemble in terms of their presentations, i.e. using treatment for a similar illness to treat similar new illnesses without known treatments of their own. Only few respondents believed that T&CMs treat and cure cancers. Majority of respondents would provide T&CMs as adjunctive therapies to patients including those with cancers. They said conscientious use of T&CMs are critical in healing of patients on conventional therapies because the T&CM and traditional healing rituals help to restore their psycho-socio-physio-anatomic equilibrium, making them become at peace within themselves, and with their fellow humans and the spirits of their ancestors in the world of the living dead [59, 73, 74]. The ancestors are perceived to play key roles in protecting and supporting their living relatives and are central to healing in traditional health practices [75].

The traditional health practitioners in this study were predominantly male and were aged above 50 years. This is mainly because the healing status was often acquired overtime through apprenticeship. Most healers are therefore older members of the society. Our finding of older THPs who are predominantly male is similar to findings from other studies from Africa and Asia: In Senegal, a study that evaluated the socio-demographic characteristics of 20 THPs engaged in management of diabetes and how they interact with patients and the community, showed that they were aged 22 – 70 years, and predominantly male (18/20). Majority (13/20) became healers through apprenticeship by family members, while five reported they received healing powers through dreams [76]. In Nigeria, most healers (70%) were aged 50 years and above [77]. Similarly, a study that involved 42 healers from Kumasi Ghana regarding their roles in cancer management showed that majority (66.7%) were males (28/42) and aged 26 – 78 years [78]. In Malaysia, a study that explored perceptions of 25 THPs on cancer and cancer prevention showed that majority (80%) were older than 30 years of age (range 26 – 68 years), and were male (80%; 20/25), and 84% were married [79]. In Guatemala, most healers were male (58.5%) and older (aged 33 – 83 years) [80]. Learning through apprenticeship requires a long period of time to master the tasks and perform well. It is therefore not surprising that THPs are older members of their societies. They bring in their experiences to bear on their roles as counsellors. The male predominance perhaps relates to the patriarchal nature of most societies which accord positions of great responsibilities to men [81–83]. The healing role is held in high esteem. To retain the healing culture in the clan, male members needed to take on the healing roles. Otherwise, the female members who get married away from the clan would go away with the healing power if this was left to the women. Women married to a particular clan could become healers, and have in some instances been healers. However, whenever marriages fail and the family separates, the woman would go away with the clan’s healing power.

Most of our respondents perceived cancer as a new and challenging disease. One respondent thought that cancers are due to an imbalance in the body. Cancers manifest themselves in some people at some point in their lives, usually when some things have gone wrong in the physical body, and or social and spiritual domains. This metaphysical conception of cancers tells in part the complexities that the disease has presented to the traditional health system, and has implications for the guidance the healers provide to patients suspected to have cancers. The respondents underscored that T&CMs and cultural rituals are required for proper healing. Our results are similar to findings from Ghana where THPs described cancer as a disease that causes swelling, wounds and destroys body tissues, spreading from one part of the body to another and that cancers occur as a result of lifestyles [78]. A study in Nigeria also showed that THPS have poor perceptions of cancers [84]. In general, the perceptions of cancers have varied among healers. Healers in Guatemala perceived cancer as a disease that is difficult to treat and cure, and which spreads through blood to many parts of the body and weakens the patient; it is a disease that causes the flesh to rot. The healers maintained that successful treatment of cancers requires restoring equilibrium in the physical, mental, emotional and spiritual sphere of life of the person and broader society [80]. These findings underscore the need for a training intervention to the THPs so they get to understand cancers better. Perhaps that will inform their decisions and treatment choices.

In this study, the perceived causes of cancers included chemical agents, accumulation of metallic residues e.g. steel wire from foods in the body, poor diets, heredity, annoyance of the spirits, accumulation of dirt in the genital tract and sexual transmissions. Some of these perceived causes, for example heredity, chemical agents from factories, and poor diets correspond to the biomedical risk factors for cancers. Such common understanding can be leveraged on to coin messages to promote cancer prevention, promote health-seeking and early detection. THPs are close to the people, and could be effective agents to educate the population on health risks and prompt health-seeking through referral to biomedical facilities. The THPs could be empowered and motivated through training and favorable collaboration agreements so they become effective and efficient without perpetuating misinformation. Our findings about perceived causes of cancers are similar to results from other studies involving traditional healers. In Nigeria, healers reported evil spirits, disrespecting cultural norms e.g. incest, and infections as causes of cancers in a person [77]. Another study in Nigeria showed that most THPs do not know the actual causes of cancers and associated cancer development and occurrence to poor diets, spirits attacks, and poor personal hygiene [84]. In Guatemala, Maya healers (67 of them) believed that cancer is a disease that is due to both material factors including consumption of harmful modern foods, frequent deliveries and sexually transmitted infections, and spiritual factors including annoying supernatural powers, and disrespecting natural elements and cultural norms, or are sent by enemies [80]. Cancer therefore is perceived within the context of the cultures of the people. Cancer control programs could therefore encompass socio-cultural beliefs about causes. For example, if the patient is content that a spiritual factor contributed to their cancer, and need spiritual healing, the spiritual healers could be allowed to do their work within the precinct of the oncology unit. “Blessing the chemotherapy” by the THPs so that it has power against the spiritual factor that caused the cancer, and so that the chemotherapy does not cause undue side effects are practices that do not undermine the value and efficacy of the chemotherapy agents, and yet incorporates the patients’ beliefs, and therefore provides them satisfaction with the treatment regime. The T&CMs and rituals help to reconcile the sick individual with his/her culture and the supernatural domain. Traditional rituals and T&CMs deal with the issues underlying the illness and sets a firm stage for the healing process. The rituals help to resolve internal conflicts within the sick person. The respondents in this study reported that such internal conflicts often hinder healing. Regarding cancer prevention, traditional healers who believe that cancers are caused by bewitchment and or resulting from the annoyance of spirits of elders long dead stressed that cancers cannot be prevented as the events that lead to cancer development are outside the locus of control of the people affected. Some THPs however belief that cancers can be prevented with use of some concoctions that are drunk by people who have not yet developed cancers [60]. These perceptions and beliefs are critical in development of public health messages to promote cancer prevention practices by aligning the biomedical messages to the beliefs in order to increase their acceptability and uptake.

Most healers in this study do not treat cancers but would refer cancer patients to their colleagues who treat cancers, and to biomedical facilities. This finding is similar to reports from Nigeria where only 8 of 20 healers said they often treated cancers [77]. A relatively higher proportion of healers in Guatemala (51.7%) reported they had ever treated patients with definitive diagnoses of cancers from hospitals [80]. In Ghana, most healers reported that they would refer their patients to biomedical care if their treatments were unsuccessful, or when they determined upfront that they would not be able to manage such a patient [76]. THPs treat cancers based on their perceived causes. Cancers that are due to an external cause i.e. non-organic are treated by methods other than herbs. These include use of divinations and magic. Cancers believed to be caused by internal factors are treated majorly with herbs. Knowledge of the right herbs come from revelations, dreams, and training through apprenticeship [85]. It is critical to health planners to not ignore the relatively high proportions who treat cancers and who could contribute to delayed health-seeking and advanced stage cancers if and when their T&CMs do not work on the cancers. A model of medical pluralism with cooperation between the mainstream biomedical health system and traditional health practices could promote patient autonomy and prompt care. The predominant paternalistic approach of mainstream biomedicine that views traditional health practices as opposition seems futile as patients will always continue to seek care with THPs either on patients’ own volitions or through persuasion by the THPs. Integration of traditional health practices into biomedicine has been advocated by many researchers and health policy makers; this approach may in the long run fail because in its core, there is an unstated intent towards killing traditional health practices by validating it using the language, tenets, philosophy and methods of biomedicine.

## Limitations

We acknowledge limitations inherent in the design. While a qualitative approach was suitable to answer our study questions, the approach does not allow for generalization and direct transferability of the findings. We endeavoured to mitigate this limitation by recruiting from both East and West Acoli so the views might be encompassing THPs of this ethnic group and promote transferability to other communities. Second, the first author is an ethnic Acoli scholar and clinician with interest in cancer early detection and integration of biomedicine with traditional health practices and or promoting medical pluralism i.e. officially recognizing and validating traditional health practices based on its own philosophies and methods rather than biomedical methods and language. Any preconceived views were however tempered by the last author who is of different ethnicity (Bantu) and training experiences. The authors regularly discussed, supplemented, contested and tempered their views and possible influences on interpretations of the data. Respondents’ validation was conducted with eight respondents because of limitation of time and financial resources. We did not discover any information and or circumstances where respondents significantly disagreed with their prior information. We therefore believe that member check was adequate and data represents the views of the respondents.

## Conclusion

Respondents consider cancers a new and challenging disease for which they have limited knowledge regarding causes and effective treatments. They believe that there could be yet to be identified effective T&CMs that treat and cure cancers. Training of THPs and the public on cancers therefore does not simply require a set of facts about cancer causes and symptoms, and the benefits of biomedical therapy, but rather messages that seek to meaningfully and respectfully confront, reverse and replace undesirable pre-existing perceptions and beliefs while promoting pre-existing helpful perceptions. The findings of this study can inform the development of targeted awareness and training interventions for THPs geared at promoting prompt symptoms recognition, health-seeking and early detection of cancers as well as prompt referral of patients with suspected cancers to the biomedical facilities. Promoting medical pluralism rather than opposition and or integration of traditional health practices could constitute a suitable model for the prompt care and improvement in outcomes for cancers and other chronic diseases, as well as other diseases.

## Supplementary Information

**Additional file 1.**

## Data Availability

The datasets used and/or analyzed during the current study available from the corresponding author on reasonable request.
