# Supplementary material for: From their own perspectives: a qualitative study exploring the perceptions of traditional health practitioners in northern Uganda regarding cancers, their causes and treatments
Source: BMC Fam Pract. 2021 Jul 19;22:155. doi: 10.1186/s12875-021-01505-w (PMC8287672; doi:10.1186/s12875-021-01505-w)
Supplement: Supplementary file 1 — Additional file 1. [file 12875_2021_1505_MOESM1_ESM.docx]

**From their own perspectives: a qualitative study exploring the perceptions of traditional health practitioners in Northern Uganda regarding cancers, their causes and treatments**

Amos Deogratius Mwaka^1^* Jennifer Achan^2^, Winnie Adoch^2^, Henry Wabinga^3^

1. Department of Medicine, School of Medicine, College of Health Sciences, Makerere University, Uganda

2. School of Public Health, College of Health Sciences, Makerere University, Uganda

3. Kampala Cancer Registry, Department of Pathology, School of Biomedical Sciences, Makerere University, Uganda

*Corresponding author: Amos Deogratius Mwaka. Department of Medicine, School of

Medicine, College of Health Sciences, Makerere University P.O Box 7072, Kampala,

Uganda. Email: [mgratius@gmail.com](mailto:mgratius@gmail.com)

**Study Guide for Traditional Health Practitioners**

**Study objectives:** Assessment of beliefs and knowledge of traditional health practitioners

**Preparations:** Inform in writing the participants for this study.

**Participants:** Traditional health practitioners (THPs).

**Time:** The interview is expected to last approximately 45 to 90 minutes.

**Informed consent:** Purpose of interview will be explained again and information sheet provided to prospective participants who will then sign an informed consent before interview commences.

**Venue:** A quiet, confidential room with no non-participants and minimal interferences.

**Recordings:** Use the attached forms to record consent and particulars of the participants. Audio recordings will be done in addition to field notes.

**Introduction:** Majority of cancer patients in Uganda are diagnosed when they are in advanced stage of disease and often experience poor treatment outcomes. We would like to discuss with you issues regarding the use of traditional and complementary medicines, the roles of traditional health practitioners and traditional practices in diagnosis, referral and treatment of breast and cervical cancers. Our main interest is to generate evidence to inform development of targeted interventions regarding the traditional health practitioners and indigenous medicine practices that may promote timelier diagnosis of breast and cervical cancers. We will highly appreciate your honest contribution to this goal.

**Understanding the roles of traditional and complementary medicines and practices in breast and cervical cancer control and care in northern Uganda**

**Participants: The traditional Health Practitioners (THPs)**

**Theme 1: Becoming a Traditional Health Practitioner or Lead healing rituals in Acholi**

1. Kindly share with us how one becomes a THP. How do people obtain healing power and hence become traditional health practitioners?

2. How did you become one? How did you obtain your healing power?

3. How is healing power passed on from one person to another or generation to generation?

4. What do you need or have to do so that your practice is better and you become more successful and more famous than others?

**Theme 2: Recognition and diagnosis of illnesses in Acholi**

1. Diagnosis of ill health in Acholi. Please tell us how illnesses were/are recognized, how a particular illness was/is confirmed, and how misfortunes (kec kom) are diagnosed.

2. Kindly tell us the different ways in which people contract illnesses or become sick in the context of the Acholi people.

Probe about infections, natural causes, spirit causes (people/elders that died long ago), curses, sorcery, and misfortunes.

3. How do the people (THPs and ritual leaders) who diagnose illnesses differentiate the causes of illnesses or sicknesses and hence select or recommend appropriate methods of treatment and healing?

**Theme 3: Illnesses that you treat**

1. Kindly tell us (list) some of the illnesses that you commonly treat.

2. How do healers specialise or decide to treat particular illnesses and not other illnesses? How do you decide which illnesses to treat and which not to treat?

3. Does your duration of practice play a role in determining the range of illnesses you treat?

4. Why did you specialize in treating those illnesses you have listed above?

**Theme 4: Healing in Acholi**

1. There are various ways of dealing with illnesses in Acholi.

(a) Please tell us some of the ways in which illnesses were/are treated. Probe about methods of treatment or treatment modalities (medicines, operations, rituals e.g. prayers, incantations).

(b) Surgery in Acholi, please tell us;

- the terms for the healers who operate,
- rationale for and processes of surgery,
- illnesses to be cured by operations,
- sterilization processes and why considered important

2. Types of THPs by names and rationale for differentiation. What underlies the different types?

**Theme 5: Opinion regarding western medicine practices**

1. Sometimes health care professionals (HCPs) tell us they do not know the causes of some cancers. What is your opinion about such responses from HCPs?

2. When you fall sick where do you go to seek care?

3. Would you recommend your family members to seek for cancer checking or screening in the medical facilities?

4. What would you wish health development partners or government to do to support you so that you can help in cancer control, prevention and care (let them list).

**Only for THPs who said they treat cancers**

**Theme 6: Cancer early detection**

1. In your opinion, what does it mean to detect or diagnose cancers early?

2. You could share with us your thoughts about what need to be done to have cancers detected early.

3. What could be some of the benefits of early detection/diagnosis of cancers?

**Theme 7: Use of traditional and complementary medicines in cancer**

1. We are interested in your opinion regarding use of traditional health practices in treatment of cancers especially breast and cervical cancers. Kindly share with us your opinion on this matter.

2. If you consider the use of traditional health practices as useful in treatment of cancers, please tell us some of the benefits of using these remedies in treating cancers.

3. Please tell us some of the indigenous and complementary medicines (the names and how they are used – roots, leaves etc.) that are used in the treatment of cancers.

Or

4. If you consider traditional remedies as not useful or even harmful in treatment of cancers, especially breast and cervical cancers, please share with us what you consider as harms or disadvantages of using these remedies in treatment of cancers.

5. What are cancers called in Acholi language (names in Luo)?

6. What causes cancer?

7. Specifically tell us the things that cause breast and cervical cancers?

Name of Interviewer: . . . . . . . . . . . . . . . . . . . . . . . . . . . . . . . . .

Signature: . . . . . . . . . . . . . . . . . . . . . . . . . . . . . . . . . . . . . . . . . . . . . . . . .

Date of interview: . . . . . . . . . . . . . . . . . . . . . . . . . . . . . . . . . . . . . . .
